# Molecular Insight into the Binding of Astilbin with Human Serum Albumin and Its Effect on Antioxidant Characteristics of Astilbin

**DOI:** 10.3390/molecules27144487

**Published:** 2022-07-13

**Authors:** Xiangyu Han, Jing Sun, Tianmei Niu, Beibei Mao, Shijie Gao, Pan Zhao, Linlin Sun

**Affiliations:** 1 College of Pharmacy, Shandong University of Traditional Chinese Medicine, Jinan 250355, China; hanxiangyujiayou@163.com (X.H.); 13065016960@163.com (J.S.); niutianmei123@163.com (T.N.); maobeibei89@163.com (B.M.); 2 School of Chinese Materia Medica, Beijing University of Chinese Medicine, Beijing 100029, China; 3 Experimental Center, Shandong University of Traditional Chinese Medicine, Jinan 250355, China; gsj77@126.com

**Keywords:** astilbin, human serum albumin, multi-spectroscopic, molecular docking, molecular dynamics simulation, anti-oxidation

## Abstract

Astilbin is a dihydroflavonol glycoside identified in many natural plants and functional food with promising biological activities which is used as an antioxidant in the pharmaceutical and food fields. This work investigated the interaction between astilbin and human serum albumin (HSA) and their effects on the antioxidant activity of astilbin by multi-spectroscopic and molecular modeling studies. The experimental results show that astilbin quenches the fluorescence emission of HSA through a static quenching mechanism. Astilbin and HSA prefer to bind at the Site Ⅰ position, which is mainly maintained by electrostatic force, hydrophobic and hydrogen bonding interactions. Multi-spectroscopic and MD results indicate that the secondary structure of HSA could be changed because of the interaction of astilbin with HSA. DPPH radical scavenging assay shows that the presence of HSA reduces the antioxidant capacity of astilbin. The explication of astilbin–HSA binding mechanism will provide insights into clinical use and resource development of astilbin in food and pharmaceutical industries.

## 1. Introduction

Flavonoids are widespread throughout the plant kingdom [1], most of which are classified into six subclasses: flavonols, flavones, flavanones, isoflavones, flavonones and anthocyanidins [2]. Studies indicate that flavonols could reduce the risk of chronic illnesses, and some have antioxidant, anti-inflammatory, anti-diabetic or neuroprotective properties [3]. Astilbin (Supplementary Materials, Figure S1) is a dihydroflavonol rhamnoside found in numerous natural plants, such as *Hypericum perforatum* [4] and *Smilax glabra* Roxb [5]. Meanwhile, astilbin is prevalent in blades and veins of healthy grapevine leaves [6]. Noteworthily, wines contain high levels of astilbin [7], which has been shown to contribute notably to the sweet taste of dry wines [8]. Modern pharmacological studies have shown that astilbin has various pharmacological activities such as anti-inflammatory [9], immunosuppressive [10], antioxidant [11], anti-diabetic [12] and cardiovascular protection [13]. Overall observations from data suggested that astilbin is a promising compound and a “star material” for the development of new health food products.

Human serum albumin (HSA) is the most important plasma proteins in the human circulatory system, with amazing ligand-binding capacity which act as a warehouse and transporter of many endogenous and exogenous compounds [14]. Hence, HSA is an important biomolecule in distribution and transport of drugs, hormones, nutrients, metabolites etc. [15]. The mature HSA has a heart-shaped three-dimensional crystal space structure with three structural domains (I−III), each with two subdomains [16], A and B, respectively. The two well-known binding sites available on HSA are Sudlow site I and II, located in subdomain IIA and IIIA [17]. A pivotal step in the domain of drug discovery is the investigation of the pharmacokinetics and pharmacodynamics of drugs [18]. The abundance of HSA makes it an important factor in the pharmacokinetic behavior of many drugs as it affects their efficacy and delivery rate, and the binding of a drug to HSA is a critical factor determining its pharmacological profiling and distribution [19]. After entering the body and binding to HSA, the small drug molecule is transported to the target site and then exerts physiological activity in the body. All these characteristic features have stimulated many efforts to understand HSA/drug binding. Astilbin has more physiological activity and medicinal value. To the best of our knowledge, there are only three studies that report the binding of astilbin with HSA or bovine serum albumin (BSA) [20,21,22]. However, their study was not comprehensive and did not address the key factors that drive the binding of drug and serum albumin. Furthermore, the effect of HSA on the biological activity of the astilbin, such as antioxidant capacity, was also not characterized.

In the present study, the lone intrinsic Tryptophan (Trp214) of HSA has served both as a solvatochromic fluorophore in fluorescence quenching measurements and as an energy donor in FRET experiments which, assisted by molecular modelling and free energy calculations, allowed it to establish the formation of a specific HSA–astilbin complex and to provide information about the structure of this complex. Hence, the interaction of HSA with astilbin was investigated systematically at the molecular level under simulated physiological conditions. Multi-spectral methods including fluorescence spectroscopy, UV-Vis, micro-FTIR and circular dichroism spectra were employed to explore the binding mechanism of astilbin to HSA as well as the conformational changes of HSA. Meanwhile, the molecular docking technique was used to visualize the location, type of force and binding energy of drug small molecules and proteins in order to determine the binding mode of astilbin and HSA at the molecular level. Dynamics simulations were applied to evaluate the dynamics and stability of the astilbin–HSA complex, and to corroborate the results obtained from spectroscopy studies. Moreover, considering the fact that oxidative stress plays an essential role in aging process [23], and astilbin exerts significant antioxidant and anti-inflammatory effects against various autoimmune diseases [24]. The effect of HSA–astilbin interaction on the 1,1-diphenyl-2-picrylhydrazyl (DPPH) free radical-scavenging activity of astilbin was also evaluated [25]. The neutralization DPPH test is based on donating electrons from the antioxidants in order to neutralize the DPPH radical, which has obvious advantages, such as ease of performing experiments, reproducibility and applicability at room temperature [26]. It can help to elucidate the binding mechanism of astilbin and HSA, and it may lay the foundation for the development of astilbin products that contribute to human health and food safety.

## 2. Results and Discussion

### 2.1. Fluorescence Quenching Studies

**Figure 1:**
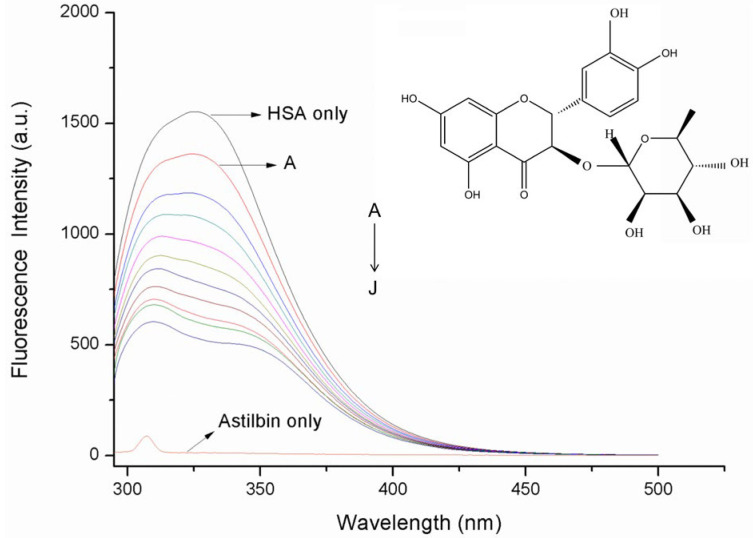
Emission spectra of HSA in the absence and the presence of astilbin, from A to J, C_HSA_: 2.5 × 10^−6^ mol·L^−1^, C_astilbin_ (A–J): 2.5, 5, 7.5, 10, 12.5, 15, 17.5, 20, 22.5, 25 × 10^−6^ mol·L^−1^, T = 298 K, λ_ex_ = 280 nm, pH = 7.4. The concentration of native astilbin was 2.5 × 10^−6^ mol·L^−1^.

HSA contains three intrinsic fluorophores, tryptophan (Trp), tyrosine (Tyr) and phenylalanine (Phe) residues [27]. HSA can emit endogenous fluorescence due to the presence of amino acid residues. The fluorescence spectra of HSA in the presence of increasing concentrations of astilbin were recorded and are represented in Figure 1. HSA exhibited an intense emission peak at 327 nm upon excitation at 280 nm. The intensity of this peak decreased obviously with increasing concentration of astilbin, accompanying a slight red shift, indicating that astilbin reacted with HSA to produce a non-fluorescent complex, which caused the fluorescent group to quench, and the fluorescence intensity decreased gradually. Meanwhile, the microenvironment in which Trp and Tyr residues in HSA were located was changed by astilbin.

The fluorescence quenching mechanism can be divided into static quenching and collisional quenching [28]. For dynamic quenching, the quenching constant increases with increasing temperature. On the opposite side, static quenching will decrease the quenching rate constants with increasing temperature. In order to explore the quenching mechanism involved in the HSA–astilbin complex and calculate the quenching constants of HSA induced by astilbin, the fluorescence intensities of HSA in each sample and the corresponding concentration of astilbin were fitted to the Stern–Volmer equation [29], which is as follows:

(1) $$\frac{F_{0}}{F}=1+K_{\mathrm{sv}}\left[Q\right]=1+k_{q}\tau_{0}\left[Q\right]$$

where F_0_ and F denote the fluorescence intensity of HSA in the absence and presence of astilbin, K_sv_ is the Stern–Volmer quenching constant, the size of its value reflects the degree of exposure of the quencher to the fluorescent material and the reaction speed, [Q] is the concentration of the quencher, τ_0_ is the average lifetime of protein in the absence of the quencher and its value for HSA is 10^−8^ S and K_q_ is the quenching rate constant of biomolecule, which can be obtained by K_sv_/τ_0_.

**Figure 2:**
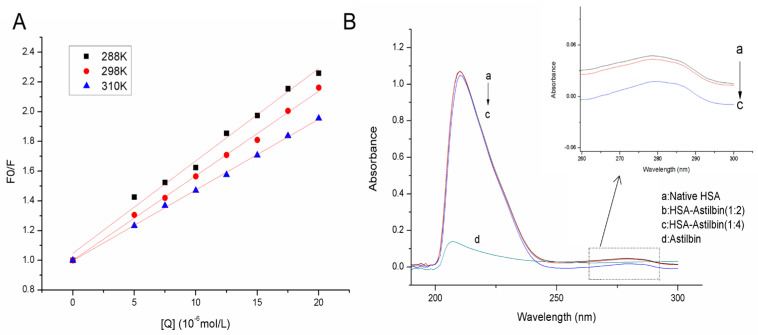
(**A**) Stern-Volmer plots for the astilbin−HSA system at three temperatures; (**B**) UV-Visible absorption spectra of HSA in the absence and presence of astilbin. C_HSA_: 1.0 × 10^−6^ mol·L^−1^. C_astilbin_ (a→c): 0, 2.0, 4.0 × 10^−6^ mol·L^−1^. Inset: the absorption spectra of HSA varied between 260 and 300 nm.

At three temperature conditions, Stern–Volmer plot of HSA–astilbin complex is shown in Figure 2A. Table 1 lists the values of quenching constant K_sv_, the quenching rate constant K_q_ and the correlation coefficient R. From the slope of this linear plot, the K_sv_ values were decreased with the increase of temperature. Meanwhile, the quenching mechanism is judged by K_q_. The K_q_ magnitude reached over the maximum diffusion constant of collisional quenching (2.0 × 10^10^ L·(mol·s)^−1^). Based on the obtained results, it could be deduced that the fluorescence quenching of HSA induced by astilbin followed static process and quenching was the result of the formation of a ground state complex between HSA and astilbin.

### 2.2. UV-Vis Absorption Spectrum

UV-Vis absorption measurement, a very simple way, is used to explore the structural change and the compound formation. In this experiment, UV-Vis absorption spectra were used to determine whether the conformation of astilbin and HSA changed upon interaction, and to verify the mechanism of fluorescence quenching. From Figure 2B, the absorbance intensity of HSA decreased obviously, and the UV-absorption spectrum was changed at 200 nm–300 nm with increasing concentration of astilbin; meanwhile, an apparent red shift was observed. The decrease and red shift of the absorption indicate that the binding of the drug induces the loosening and unfolding of the protein skeleton and decreases the hydrophobicity of the microenvironment of the aromatic amino acid residue, further indicating that the fluorescence quenching mechanism of astilbin on HSA was a static process [30].

### 2.3. Binding Constant and the Number of Binding Sites

For the static quenching process, the binding constant and the number of binding sites for the HSA and astilbin interaction were obtained with the double logarithmic curve represented by the following equation [31]:

(2) $$\mathrm{lg}\left(F_{0}-F\right)/F={\mathrm{lgK}}_{a}+\mathrm{nlg}\left[Q\right]$$

where K_a_ and n represent the binding constant and the number of binding sites; F_0_ and F denote the fluorescence intensities of HSA in the presence and absence of astilbin, respectively. [Q] is the concentration of astilbin.

**Figure 3:**
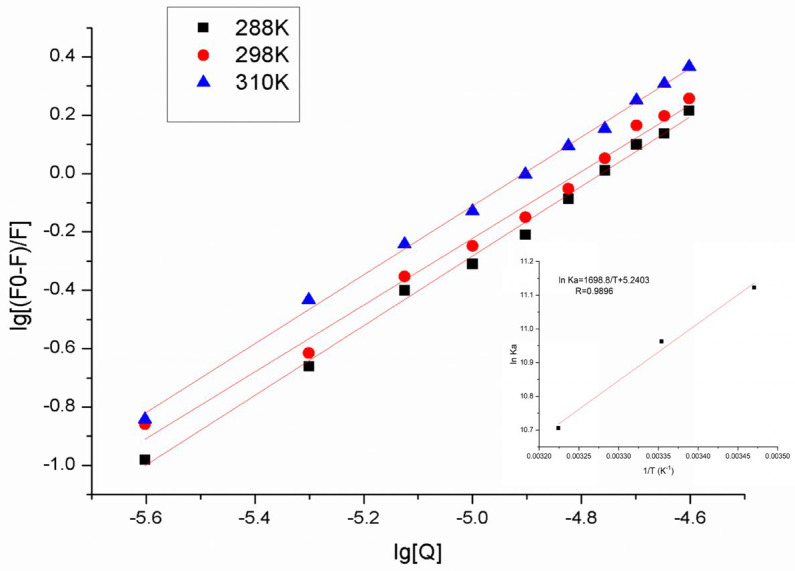
Scatchard diagram of astilbin–HSA system at different temperatures. The insert corresponds to the Van’t Hoff plot for the interaction of astilbin and HSA.

A fitted straight line was obtained by mapping the lg [Q] with lg [(F_0_ − F)/F] (Figure 3). The slope of the double logarithmic regression curve provided the number of binding sites, whereas the intercept was used to calculate the binding constant. The corresponding binding constants of HSA–astilbin system in three different temperatures were summarized in Table 2. The values of K_a_ were approximately equal to 10^4^ M^−1^, which suggested moderate binding interactions between HSA and astilbin, since the binding affinity of chemicals with transport proteins could affect their biological activity. Generally, drugs with strong protein binding have long half-life, long duration time and slow work and elimination in plasma [32]. The values of n were close to 1, which suggested the presence of one binding site in HSA for astilbin. Moreover, the binding constant K_a_ of the interaction between HSA and astilbin decreased gradually with the increase of temperature, and the number of binding sites n also decreased, indicating that the temperature has an influence on it.

### 2.4. Thermodynamic Interaction

The thermodynamic parameters include the enthalpy change (ΔH^0^), entropy change (ΔS^0^) and Gibbs free energy (ΔG^0^). The thermodynamic parameters can be calculated from the binding constants K_a_ at different temperatures, according to Van’t Hoff plot using the Van’t Hoff equation.

(3) $${\mathrm{lnK}}_{a}=-\Delta H^{0}/\mathrm{RT}+\Delta S^{0}/R$$

where K_a_ is the binding constant at the corresponding temperature and R is the gas constant, R = 8.314 J·mol^−1^·K^−1^. T indicates the absolute temperature of the experimental process, 288 K, 298 K and 310 K, respectively.

The enthalpy change (ΔH^0^) could be obtained from the slope of the Van’t Hoff plot. The free energy change is calculated from the following relationship (Gibbs free energy equation):

(4) $$\Delta G^{0}=\Delta H^{0}-T\Delta S^{0}=-{\mathrm{RTlnK}}_{a}$$

As shown in Figure 3, a well-fitted linear line can be obtained by plotting. The ΔH^0^ and ΔS^0^ are obtained from the slope and intercept of Van’t Hoff curve, respectively. The values of ΔH^0^, ΔS^0^ and ΔG^0^ at different temperatures are presented in Table 2. The positive value of ΔS^0^ (43.57 kJ·mol^−1^) demonstrated that the hydrophobic forces and electrostatic force may be the dominant forces [33]. Due to the complexity of HSA, there are often synergistic effects of multiple forces, rather than a single force acting alone. Therefore, the role of hydrogen bonding should also be considered in the binding process. The negative values of ΔG^0^ suggested the spontaneity of binding process between astilbin and HSA.

### 2.5. Determination of Energy Transfer

Fluorescence resonance energy transfer (FRET) has been employed to measure the molecular binding distance of small molecule drugs to HSA. Referring to Förster’s non-radiation energy transfer theory, the average distance related to the efficiency of energy transfer (E) from donor to acceptor can be calculated from the following equation:

(5) $$E=1-\left(F/F_{0}\right)=R_{0}{}^{6}/(R_{0}{}^{6}+r^{6})$$

where F_0_ and F are the fluorescence intensities of HSA in the presence and absence of astilbin, respectively. r is the binding distance between astilbin and HSA. R_0_ is the critical distance at 50% energy transfer efficiency, whose value can be obtained from the following equation:

(6) $$R_{0}{}^{6}=8.78\times 10^{-23}k^{2}n^{-4}\Phi J$$

where k^2^ is the spatial orientation factor of the dipole leap between astilbin and HSA; n is the average refractive index of medium; Φ is the quantum yield of HSA; J is overlap integral of the donor emission spectrum and the acceptor absorption spectrum that can be determined by the following equation:

(7) $$J=\frac{\sum^{}F\left(\lambda \right)\varepsilon \left(\lambda \right)\lambda^{4}\Delta \lambda }{\sum^{}F\left(\lambda \right)\Delta \lambda }$$

where F(λ) is the fluorescence intensity of HSA when wavelength equals to λ, ε(λ) is the molar absorbance coefficient of astilbin at the wavelength λ and Δλ is the wavelength span separated in the calculation of the overlap integral.

At 298 K, the UV-Vis and fluorescence spectra of HSA–astilbin was obtained under the same detection conditions as described earlier. Figure S2 exhibited the overlap between the fluorescence emission spectrum of HSA and the UV-Vis absorption spectrum of astilbin. As a rule, the values of k^2^, n and Φ are 2/3, 1.336 and 0.118. Consequently, the values of J, E, R_0_ and r were determined as 6.51 × 10^−11^ cm^3^·L·mol^−1^, 0.1574, 2.3749 nm and 3.1411 nm. Apparently, according to Förster’s non-radiation energy transfer theory [34], the binding distance between the tryptophan residue in HSA and the astilbin molecule is 3.1411 nm, which is less than 7 nm and 0.5 R_0_ < r < 1.5 R_0_, indicating that the energy transfer between astilbin and HSA occurred.

### 2.6. Identification of Binding Location

According to the results of Section 2.3, there was probably one single binding site for the HSA–astilbin complex. The two well-known binding sites available on HSA are Sudlow site I and II, located in subdomain IIA and IIIA (Figure S3A). In this study, warfarin sodium and ibuprofen were used as site markers for Site I and Site II [35]. The equal molar concentration for HSA and either of the site markers (ibuprofen or warfarin sodium) were interacted with different concentrations of astilbin, the fluorescence emission spectra of the system containing HSA–astilbin mixture were recorded. The fluorescence spectra of the system were shown in Figure S3B,C, respectively.

The addition of warfarin sodium solution to HSA decreased the fluorescence intensity of HSA to a large extent; the addition of ibuprofen to HSA dropwise decreased the fluorescence intensity of HSA to a lesser extent. The experimental results suggest that the binding site of astilbin and HSA may be Site Ⅰ.

Moreover, to quantify the site competition experiments between astilbin and HSA, the Stern–Volmer equation and the double logarithmic regression curve were used to calculate the changes in the binding constants K_a_ of the astilbin–HSA system in the presence and absence of the two markers under the same conditions. The binding constant recorded in presence of warfarin was found to decreased significantly by 76.65% and in presence of ibuprofen was not significantly different from that of the system without the marker. The binding constant for the interaction in which site I marker warfarin was used was lower than the binding constant obtained in presence of ibuprofen or without site marker as given in Table 3. The results suggest the replacement reaction between astilbin and warfarin sodium occurred and site I of HSA as the possible binding site for interaction.

### 2.7. Effect of Astilbin on the Conformation of HSA

#### 2.7.1. Synchronous Fluorescence Spectroscopy Studies

Synchronous fluorescence spectroscopy is a widely used method to study the microenvironment in which amino acid residues are located. Synchronous fluorescence spectroscopy technique was used to identify micro-environmental changes in the fluorophore amino acid residues Trp and Tyr [36]. A shift in the emission wavelength suggests change in the polarity around the fluorophore residues [37]. The amino acid tyrosine’s micro-environmental information is obtained at ∆λ = 15 nm, whereas that for tryptophan is obtained at ∆λ = 60 nm. The synchronous fluorescence spectra are presented in Figure S4A,B.

**Figure 4:**
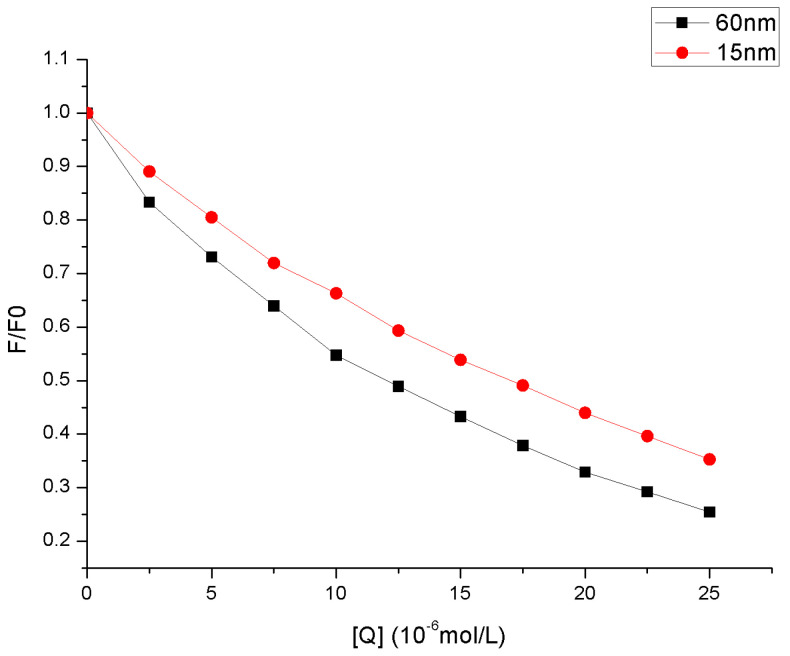
The quenching of HSA synchronous fluorescence by astilbin; C_HSA_ = 2.5 × 10^−6^ mol·L^−1^. C_astilbin_ (a→k): 0, 2.5, 5, 7.5, 10, 12.5, 15, 17.5, 20, 22.5, 25 × 10^−6^ mol·L^−1^.

The fluorescence quenching at ∆λ = 15 nm (Figure S4A) which represent the tyrosine residues is low, suggesting minimum interference in the vicinity of tyrosine residues in interaction with astilbin as there is feeble decrease in the fluorescence intensity. However, an apparently higher decrease in fluorescence intensity at ∆λ = 60 nm (Figure S4B) indicates a micro-environmental change in the vicinity of tryptophan residues. The quenching slope at ∆λ = 60 nm was significantly greater than ∆λ = 15 nm, and the fluorescence quenching of the tryptophan residue was significantly greater than the tyrosine residue, as seen in Figure 4. Moreover, a small shift of 3 nm showed at ∆λ = 15 nm, whereas the shift of 5 nm was observed at ∆λ = 60 nm in the emission wavelength, indicating that the addition of astilbin would change the conformation of HSA microregion to some extent.

#### 2.7.2. Three-Dimensional Fluorescence Spectroscopy

**Figure 5:**
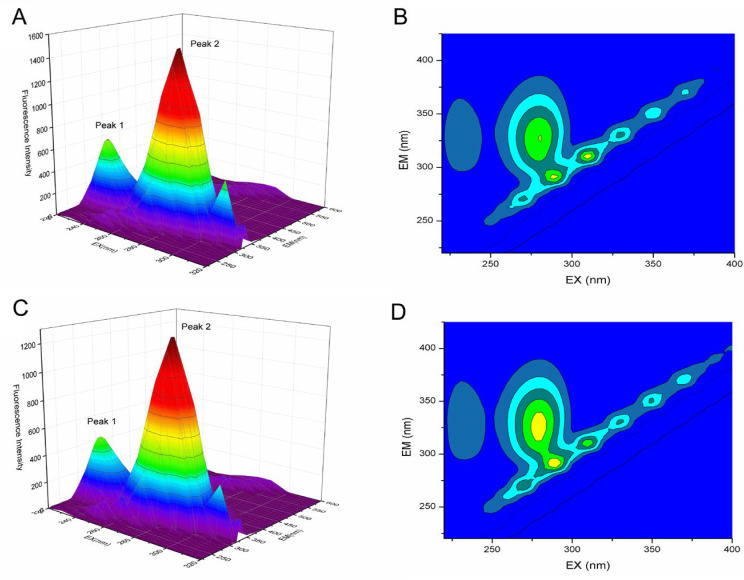
Three-dimensional fluorescence spectra and contour map of HSA (**A**,**B**) and astilbin−HSA (**C**,**D**). C_HSA_ = 2.5 × 10^−6^ mol·L^−1^; C_astilbin_ = 5.0 × 10^−6^ mol·L^−1^; T = 298 K, pH = 7.4.

The protein structural changes were accessed using three-dimensional (3D) fluorescence spectroscopy. This technique provides detailed information about the conformational changes that occur during the protein ligand interaction [38]. In the absence and presence of astilbin, the 3D fluorescence spectra of HSA and the corresponding contour plots are shown in Figure 5, and the relevant parameters are shown in Table 4. Peak 1 reveals the transition of Trp and Tyr residues, and Peak 2 exhibits the transition of the polypeptide backbone structures [39]. The intensity of Peak 1 decreased from 1539 to 1291 (16.11%) with the addition of astilbin. The blue shift in the emission peak from 330 nm to 320 nm indicated that the microenvironment polarity of Tyr and Trp residues was decreased by the interaction of astilbin and has. The fluorescence intensity of Peak 2 decreased from 623.2 to 478.6 (23.20%) with the addition of astilbin, indicating that the binding of astilbin disturbed the secondary structure of HSA. Once again, the above experimental results demonstrated the conformational change of HSA caused by the interaction with astilbin.

#### 2.7.3. Micro-FTIR Spectrum

**Figure 6:**
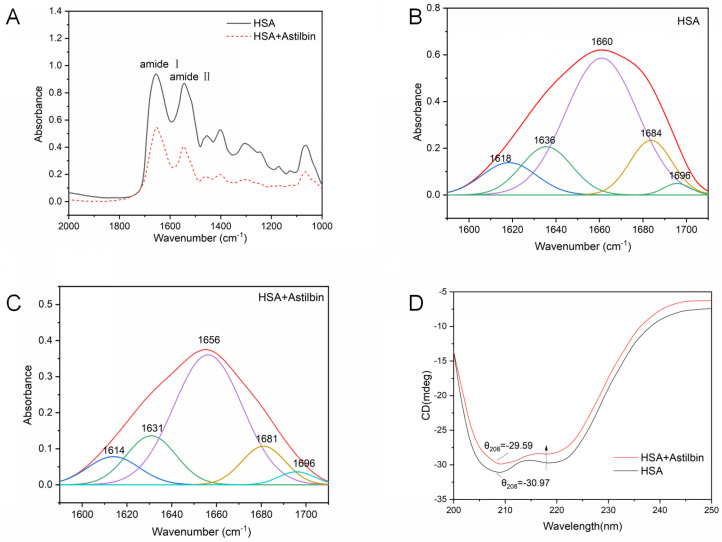
(**A**) Micro-FTIR spectra of HSA and HSA−astilbin system. (**B**) Curve fitting micro-FTIR spectra of infrared amide I band of HSA. (**C**) Curve fitting micro-FTIR spectra of infrared amide I band of HSA−astilbin system. (**D**) CD spectra of HSA and HSA−astilbin system.

Micro-FTIR spectroscopy was used to analyze the effect of the presence of astilbin on the conformational changes of HSA, and thus to explore specifically the changes in the secondary structure of the protein. The secondary structure of HSA is shown as characteristic absorption peaks on the IR spectrogram. Among them, the amide I band (1700–1600 cm^−1^), which mainly absorbs the C=O stretching vibration of amino acid residues, is very sensitive to the change of its secondary structure; the amide II band (1600–1500 cm^−1^) contains the stretching vibration of C–N and the N–H deformation vibrations, which is also an important spectral band reflecting the protein structure [40]. Figure 6A shows that the interaction of astilbin with HSA shifted the amide I band of HSA from 1654 cm^−1^ to 1650 cm^−1^ and the amide II band from 1542 cm^−1^ to 1546 cm^−^^1^, and the absorbance intensity of the amide I and II bands decreased due to the addition of astilbin.

The amide I band contains different secondary structure information, which is more valuable for protein research. HSA secondary structures include β-sheet, random coil, α-helix, β-turn and β-antiparallel [41]. By curve fitting procedure, secondary structures of the HSA−astilbin system and free HSA were estimated by analyzing the amide I band (Figure 6A–C) with their positioned FT-IR vibrations. Percentages are presented in Table 5. It indicates that the presence of astilbin caused some internal changes in the secondary structure of HSA and affected its conformation.

#### 2.7.4. Circular Dichroism Spectra

CD is a widely used means to study the conformational changes of HSA and to determine the number of α-helices [42]. HSA has two obvious negative characteristic peaks around 208 nm and 222 nm, which are typical of the α-helix structure, and these two peaks are contributed by π−π * and n−π * of α-helical peptide bonds, respectively [43].

The addition of astilbin significantly reduced the negative ellipticity of HSA at 208 nm and 222 nm, but the shape and position of the two peaks did not change significantly. It indicated that the addition of astilbin changed the secondary structure of HSA, but it was still dominated by the α-helical structure. α-helical structure content can be calculated by the following formula.

(8) $$\mathrm{MRE}=\frac{\theta_{\mathrm{obs}\left(\mathrm{mdeg}\right)}}{10\times c_{p}\mathrm{nl}}$$

(9) $$\alpha -\mathrm{helix}\left(\%\right)=\frac{-{\mathrm{MRE}}_{208}-4000}{33,000-4000}\times 100\%$$

where MRE_208_ denotes the MRE value observed at 208 nm, C_p_ denotes the molar concentration of HSA, n denotes the number of residues of HSA (n = 585), l denotes the thickness of the cuvette, 4000 denotes the MRE value of β-sheet and random coil at 208 nm and 33,000 denotes the MRE value of α-helix at 208 nm.

Figure 6D shows that the content of α-helix of astilbin−HSA complex at the molar ratio of 5:1 decreased compared with free HSA. The results demonstrate that the interaction between astilbin and HSA resulted in the conformational change of HSA and affected the stability of the α-helix for the HSA and astilbin system.

### 2.8. Molecular Docking Technology

**Figure 7:**
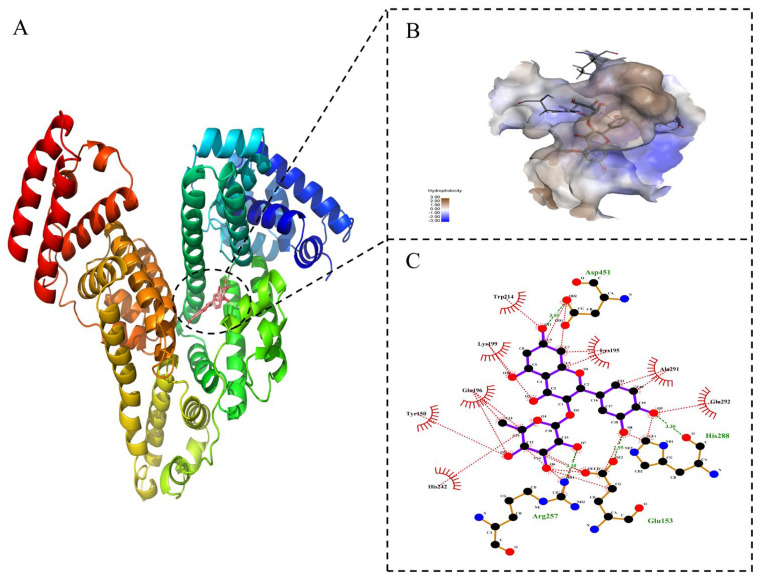
Molecular docking diagram of astilbin–HSA system. ((**A**) Molecular docking map of astilbin binding site Ⅰ on HSA; (**B**) astilbin located in the hydrophobic cavity of site I; (**C**) 2D diagram of the interaction of HSA with astilbin.).

Molecular docking is an effective method to study the optimal binding site and binding force between small ligand and biomacromolecule [44]. The molecular docking technique can visualize the location of small drug molecules acting with proteins, the type of force and the binding energy, with good visibility of the stereospecific binding pattern. To recognize the putative binding location as well as the amino acid residues near the binding site, molecular docking studies of HSA with astilbin were carried out using AutoDock Vina. The optimal binding model of astilbin–HSA is shown in Figure 7A. Astilbin tended to be bound to site I, which was consistent with the results of site competition experiments. This exhibited the lowest binding energy of −8.1 kcal·mol^−1^ (−33.91 kJ·mol^−1^), which indicated the possibility of binding.

Astilbin was inserted into the hydrophobic cavity at site I of HSA (Figure 7B). As shown in Figure 7C, astilbin was mainly surrounded by GLU−153, ARG−257, HIS−288, ALA−291, GLU−292, ASP−451, TRP−214, LYS−195, LYS−199, GLN−196, HIS−242 and TYR−150. Astilbin and these active amino acids maintained the stability of the binding conformation mainly by the hydrophobic interaction (the red dotted line) and hydrogen bonding force (the green dotted line). The above molecular docking results are in approximate agreement with the thermodynamic interaction experimental results.

### 2.9. Molecular Dynamics Simulations

**Figure 8:**
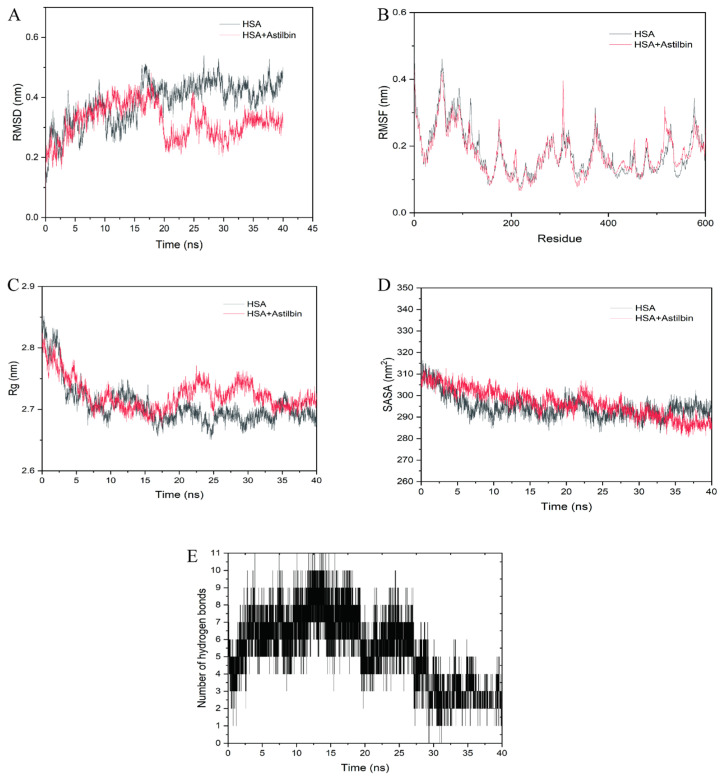
(**A**) The plots of RMSD, (**B**) RMSF, (**C**) Rg and (**D**) SASA of HSA−astilbin complex and free HSA. (**E**) The number of hydrogen bonds between astilbin and HSA during MD simulation.

Any ligand that binds to protein may induce structural and conformational changes in the macromolecule, which is very important for the pharmacokinetics and pharmacodynamics [45]. MD can be used to study the stability and kinetic characteristics of astilbin–HSA complexes [46]. The root mean square deviation (RMSD) is a parameter used to measure the stability of the system [47]. In Figure 8A, from 0 to 20 ns, the RMSD curves fluctuated widely, indicating that the skeletal structure of HSA changed during the binding process of astilbin. Furthermore, 20 ns later, the RMSD value of the complex was smaller than that of free HSA, indicating that the complex was more stable than free HSA and the binding could induce the overall mechanism to reach a stable state.

The root mean square fluctuation (RMSF) is a reflection of the structural flexibility of HSA [48]. However, at the region of binding site I, the RMSF values of the complexes in Figure 8B were mostly lower than those of free HSA, indicating that astilbin was bound stably to the binding site I of HSA and restricted the movement of residue backbone atoms to some extent. This suggested that hydrogen bonding forces and hydrophobic forces previously discussed played a key role in the stabilization of astilbin at the binding site and the stabilization of the surrounding amino acid residues. Hence, the interaction between astilbin and HSA affected the flexibility of this amino acid residue, resulting in a change in its surroundings, which in turn led to a change in the secondary structure of HSA. The radius of gyration (Rg) describes the compactness of the structure of the system [49]. The radius of gyration value of the complex decreased, and was larger than that of the free HSA in Figure 8C. It indicates that the secondary structure of the complex was slightly changed after the binding of the astilbin and HSA, resulting in the loosening of the whole amino acid helix structure and thus the secondary structure of HSA was changed.

The solvent accessible surface area (SASA) of amino acid residues in the HSA system was slightly shifted by astilbin, indicating that astilbin can affect the surface hydrophobicity of HSA (Figure 8D). Figure 8E depicts the number of hydrogen bonds in the HSA–astilbin complex as a function of simulation time. The number of hydrogen bonds fluctuates between 0 and 11 throughout the MD process. Since the atoms of HSA and astilbin are in a continuous state of vibration and change during the MD process, the movement of astilbin at the binding site leads to changes in the interaction forces between astilbin and the surrounding amino acids.

### 2.10. Effect of HSA on the Antioxidant Capability of Astilbin

Zhao [50] evaluated the antioxidant capacity of isolated astilbin, which are dihydroflavonol glycosides with antioxidant capacity. In this paper, the in vitro antioxidant capacity of astilbin was determined by the DPPH scavenging method, and the effect of HSA on the free radical scavenging capacity of astilbin was investigated.

**Figure 9:**
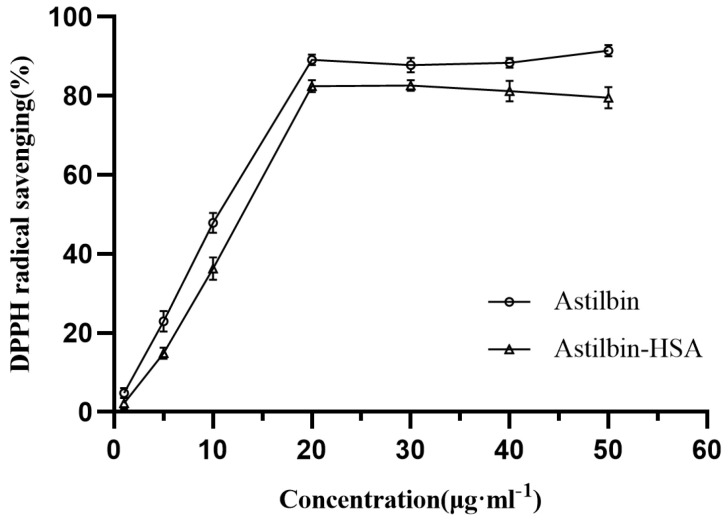
The antioxidant activity in the samples with different concentrations of astilbin (C_Astilbin_:C_HSA_ = 1:1).

From Figure 9, the DPPH radical scavenging rate gradually increased with the higher concentration of astilbin. After mixing with HSA, although the DPPH radical scavenging rate also gradually increased with the increase of astilbin concentration., it decreased compared with the DPPH radical scavenging rate before mixing. Meanwhile, the astilbin showed potent antiradical activity (IC_50_ = 9.653 μg·mL^−1^), while the astilbin−HSA had an IC_50_ of 10.68 μg·mL^−1^. This suggests that the interaction with HSA reduces the antioxidant capacity of astilbin. The reason for this phenomenon may be that during the interaction with HSA, some of the active sites of astilbin were encroached by HSA, which prevented it from binding to DPPH radicals and reduced its antioxidant capacity. At the same time, after binding to HSA, the greater spatial resistance also hinders the free binding of astilbin to free radicals.

## 3. Materials and Methods

### 3.1. Materials and Sample Preparation

HSA (70024−90−7, LOT: No. 401L057) was purchased from Beijing Solarbio Science & Technology Co., Ltd., Beijing, China; astilbin (LOT:MUST−20032410, 98.14% by HPLC) was provided by Chengdu Manstead Biotechnology Co., Ltd., Chengdu, China; all other reagents were analytical grade and distilled water was used in all experiments. The stock solution of HSA was prepared in Tris-HCl buffer solution (simulating physiological environment) at concentration of 0.05 mol·L^−1^. The stock solutions of 2.5 × 10^−3^ mol·L^−1^ astilbin were prepared in 50% methanol, and warfarin sodium and ibuprofen were prepared to make 2.5 × 10^−3^ mol·L^−1^ stock solution. The stock solutions were stored in 4 °C refrigerated in the dark. Experimental solutions of HSA and astilbin were prepared by appropriate dilution of their stock solutions.

### 3.2. Fluorescence Measurements

#### 3.2.1. Fluorescence Spectroscopy Experiments

Fluorescence measurements were carried out on a fluorescence spectrometer (Hitachi F−2700, Tokyo, Japan) at 298 K, 303 K and 310 K. Respectively, a certain concentration of 2.5 × 10^−3^ mol·L^−1^ astilbin was added into 2.5 × 10^−6^ mol·L^−1^ HSA. The concentration of astilbin gradually increased from 0 to 2.5 × 10^−5^ mol·L^−1^ with interval of 2.5 × 10^−6^ mol·L^−1^. The samples were fully reacted for 5 min, excited at 280 nm and the emission spectra were recorded at wavelengths from 220 to 500 nm. The excitation and emission slit widths were fixed at 5 nm, and the scanning speed was 1500 nm·min^−1^.

#### 3.2.2. Synchronous Fluorescence Spectroscopy

Respectively, a certain concentration of 2.5 × 10^−3^ mol·L^−1^ astilbin was added into 2.5 × 10^−6^ mol·L^−1^ HSA. The concentration of astilbin was varied from 0 to 2.5 × 10^−5^ mol·L^−1^ with the step of 2.5 × 10^−6^ mol·L^−1^. The sample’s reaction was performed for 5 min. By setting the excitation and emission wavelength intervals to ∆λ = 15 nm and ∆λ = 60 nm, the spectroscopic behavior of tyrosine (Tyr) and tryptophan (Trp) residues in the HSA was monitored at 298 K. The simultaneous fluorescence spectra in the range of 265–365 nm and 220–320 nm were scanned.

#### 3.2.3. Three-Dimensional Fluorescence Spectroscopy

Respectively, a certain concentration of 2.5 × 10^−3^ mol·L^−1^ astilbin was added into 2.5 × 10^−6^ mol·L^−1^ HSA, so that the molar concentration ratios of astilbin and HSA were 0:1 and 2:1. The samples were fully reacted for 5 min and scan the 3D spectrum. The excitation and emission wavelength were set as 220–400 nm and 220–500 nm.

#### 3.2.4. Site Marker Competitive Binding Experiments

Increasing concentrations of astilbin were added to solutions containing HSA and warfarin or ibuprofen. The molar concentration ratios of astilbin and HSA were 0:1 to 10:1. The samples were stirred for 5 min at 298 K. Then the samples were excited at 280 nm and fluorescence emission spectra were recorded at wavelengths from 220 to 500 nm.

### 3.3. Ultraviolet-Visible Spectroscopy

The ultraviolet-visible (UV-Vis) absorption spectrum was measured by employing a METASH (X−5 type) UV-Vis spectrophotometer (Shanghai Yuananalysis Instruments Co., Shanghai, China) equipped with a 1.0 cm quartz cell. The concentration of HSA was fixed at 2.5 × 10^−6^ mol·L^−1^, and the final concentration of astilbin in the samples were 0, 5 × 10^−6^, 1 × 10^−5^ mol·L^−1^. The UV absorption spectra of HSA were measured in the range of 190–350 nm at 298 K.

### 3.4. Circular Dichroism Spectroscopy

The changes in the secondary structure of HSA due to astilbin were analyzed by circular dichroism (CD) spectroscopy. The solution of astilbin and HSA solution was mixed well so that the final molar concentration ratios of astilbin to HSA were 0:1 and 5:1, respectively, and reacted for 5 min at room temperature. An appropriate amount of the above sample solution was placed in a 1 mm cell size quartz cuvette and scanned on a 4207A000 CD spectrometer (Applied Photophysics Ltd., Leatherhead, Surrey, UK). The scanning wavelength was set at 200~240 nm, the scanning speed was 100 nm·min^−1^ and the response time was 1 s. During the scanning, the sample compartment and optical path were continuously flushed with high purity nitrogen at 25 °C. The same concentration of drug solution was automatically deducted as blank background during the test.

### 3.5. Micro-FTIR Measurement

Micro-FTIR spectra of HSA and HSA–astilbin were measured by scanning on an infrared spectrometer (Thermo Scientific Nicolet iN10, Waltham, MA, USA). Micro-FTIR reflects the changes in the secondary structure of HSA. The IR spectrum of HSA solution (2.0 × 10^−6^ M) were recorded at room temperature, and then astilbin (2.5 × 10^−3^ mol·L^−1^) was added to it, so that the molar concentration ratio of astilbin to HSA was 5:1. Micro-FTIR spectra were taken with a wavelength range of 2000~1000 cm^−1^, and the resolution was set as 4 cm^−1^ with 32 scans. The infrared spectra of HSA and HSA–astilbin system were recorded after deducting the blank background.

### 3.6. Molecular Docking Simulation

Molecular docking is an effective method to study the optimal binding site and binding force between small ligand and biomacromolecule. The 3D structure of astilbin was downloaded from the PUBCHEM database and energy minimization was performed under the MMFF94 force field. The HSA structure (PDB code: 1H9Z, Resolution: 2.5 Å) was obtained from the PDB database [51], and HSA was treated with PyMol 2.5 [52], including the removal of water molecules, salt ions and small molecules. The docking box was then set up to wrap the active site. In addition, ADFRsuite 1.0 [53] was used to convert the processed astilbin and HSA into the PDBQT formats. Docking was performed using AutoDock Vina 1.1.2 software [54], and the exhaustiveness of the global search was set to 32 and the rest of the parameters were kept at their default settings. The output docking conformation with the highest score was considered as the binding conformation, and finally the docking results were visualized and analyzed using PyMol 2.5 and Discovery studio viewer.

### 3.7. Molecular Dynamics Simulations

Molecular dynamics simulations (MD) of astilbin–HSA complexes were performed using GROMACS 2019.6 to evaluate the effect of astilbin on the HSA structure. The docking results were selected as the initial structure, Amber 14SB was chosen as the force field and TIP3P as the internally filled water molecule. A water box was created and sodium ions were added to equilibrate the system. The energy for the maximum number of steps (50,000) was minimized by the steepest descent algorithm. The cut−off distances of the Coulomb force and van der Waals interactions were both 1.4 nm. MD simulations were performed for 40 ns at standard atmospheric pressure and 300 K temperature simulations [55]. After the MD simulation, the periodic boundary removal process was performed to obtain parameters in series. Root mean square deviation (RMSD), radius of gyration (Rg) and root mean square fluctuation (RMSF) were calculated by analyzing the trajectory files of the docked complex interaction (section for whole protein) [56]. Meanwhile, Solvent Accessible Surface Area (SASA) and the number of hydrogen bonds were enumerated to obtain detailed structural changes in HSA by the astilbin.

### 3.8. Effect of HSA on the Antioxidant Capacity of Astilbin

The antioxidant activity of astilbin in the presence and absence of human serum albumin was determined by measuring the scavenging rate of DPPH radicals. Human serum albumin was dissolved in Tris-HCl buffer, and astilbin and DPPH were dissolved in methanol and anhydrous ethanol to final concentrations of 1 g·L^−1^, 1 g·L^−1^, and 1.5 × 10^−4^ M. All solutions were stored as stock solutions at 4 °C in the dark, and the DPPH solution was ready to use for 3−5 h. The sample of astilbin was diluted with methanol to 1, 5, 10, 20, 30, 40 and 50 μg·mL^−1^ separately, and then 2 mL of the diluted astilbin was mixed with an equal amount of DPPH solution. For the samples of DPPH and astilbin in the presence of HSA, the astilbin stock solution was first mixed with the HSA to the above concentrations, and then equal amounts of DPPH solution were added respectively. All solutions were gently shaken and mixed, and the reaction was carried out at room temperature for 30 min protected from light. The maximum absorbance of DPPH at 521 nm was recorded using UV-Vis spectrophotometer (Shanghai Yuan analysis Instruments Co., Shanghai, China). A suitable blank was kept to eliminate the effect of ethanol on the absorbance determination. The scavenging activity of DPPH radicals was calculated according to the following equation [57].

(10) $$\mathrm{Radical}\;\mathrm{scavening}\left(\%\right)=\left(A_{\mathrm{control}}-A_{\mathrm{sample}}/A_{\mathrm{sample}}\right)\times 100$$

where A_control_ is the absorbance of DPPH alone and A_sample_ is the absorbance of DPPH with astilbin in the presence or absence of HSA.

## 4. Conclusions

This study used multi-spectral techniques and molecular docking methods to elucidate the interaction of astilbin with the model transporter protein HSA under physiological conditions. The effect of binding on the antioxidant activity of astilbin was evaluated by DPPH radical scavenging assay. Spectroscopic analysis showed that astilbin could effectively quench the endogenous fluorescence of HSA through the static quenching mechanism, and the non-radiative energy transfer between the two was also a cause of fluorescence quenching. Astilbin and HSA prefer to bind at the site Ⅰ position. The hydrophobic forces and hydrogen bonds are the main forces of the binding process. These results are also well supported by molecular docking studies. Multi-spectral methods and MD results indicate that the secondary structure of HSA could be changed because of the interaction of astilbin with HSA. In addition to micro-FTIR spectroscopy and circular dichroism illustrated that the α-helix content of HSA was reduced after binding. The antioxidant capacity of the HSA–astilbin complex was reduced compared to that of free astilbin. The reason may be that some active sites of astilbin were encroached by HSA and the spatial site resistance was greater after binding.

In conclusion, the findings of this study provide a comprehensive understanding of the interaction between HSA and astilbin, which provides a theoretical basis for the in vivo transport and metabolism of astilbin and its effect on the structure of HSA, as well as a reference for the detection of the mode of action of other flavonoids with proteins. The finding of this study may provide a meaningful experimental and theoretical basis for the evaluation of astilbin’s therapeutic potential and for future rational drug design studies.

## Figures and Tables

**Table 1:** Stern-Volmer quenching constants for the interaction of astilbin with HSA.

pH	T (K)	K_sv_ (×10^4^ L·mol^−1^)	K_q_ (×10^12^ L·mol^−1^·s^−1^)	R	SD
	288	6.44	6.44	0.9897	0.14
7.4	298	5.70	5.70	0.9980	0.12
	310	4.70	4.70	0.9991	0.09

R is the correlation coefficient. SD is the standard deviation for the K_sv_ values.

**Table 2:** Binding constants (K_a_), the number of binding site (n) and relative thermodynamic parameters of the HSA–astilbin system at three temperatures.

pH	T (K)	K_a_ (×10^4^ L·mol^−1^)	n	R	ΔH^0^ (kJ·mol^−1^)	ΔG^0^ (kJ·mol^−1^)	ΔS^0^ (J·mol^−1^·K^−1^)
	288	6.77 ± 0.15	1.02	0.9856		−26.67 ± 0.05	
7.4	298	5.77 ± 0.02	1.00	0.9985	−14.12 ± 0.54	−27.11 ± 0.01	43.57 ± 1.75
	310	4.46 ± 0.03	0.99	0.9959		−27.63 ± 0.02	

R is the correlation coefficient.

**Table 3:** Binding constants of competitive experiments in the complexation between astilbin and HSA.

pH	Site Marker	K_a_ (×10^4^ L·mol^−1^)	R
	Blank	22.74 ± 0.09	0.9957
7.4	Ibuprofen	19.93 ± 0.12	0.9932
	Warfarin sodium	5.31 ± 0.15	0.9927

R is the correlation coefficient.

**Table 4:** Three-dimensional fluorescence spectral characteristic parameters of HSA and astilbin−HSA system.

System	Peaks	Peaks Position λex/λem (nm/nm)	Stokes Shift Δλ (nm)	Fluorescence Intensity (a.u.)
Native HSA	Fluorescence peak 1	280.0/330.0	50	1539
	Fluorescence peak 2	230.0/330.0	100	623.2
Astilbin−HSA (2:1)	Fluorescence peak 1	280.0/320.0	40	1291
	Fluorescence peak 2	230.0/330.0	100	478.6

**Table 5:** Secondary structure contents analysis of HSA and astilbin−HSA system.

System		Structure Component (%)				
	α-Helix		β-Sheet	β-Turn	β-Antiparallel	Random Coil
Method	FTIR	CD	FTIR			
Native HSA	59.06 ± 0.18	59.24 ± 0.16	10.60 ± 0.14	13.86 ± 0.09	1.85 ± 0.09	14.63 ± 0.13
Astilbin–HSA	56.31 ± 0.17	55.98 ± 0.15	7.36 ± 0.08	14.01 ± 0.05	2.33 ± 0.04	20.00 ± 0.19

## Data Availability

The data presented in this study are contained within the article and Supplementary Materials.

## Supplementary Materials

The following supporting information can be downloaded at: https://www.mdpi.com/article/10.3390/molecules27144487/s1, Figure S1: The Chemical structure of astilbin. Figure S2: Overlap of the fluorescence emission spectrum of HSA with the UV-Vis absorption of astilbin. Figure S3: (A) Structure of HSA showing the subdomains; (B,C) Effects of different site competitors on HSA–astilbin complex. Figure S4: (A) Synchronous fluorescence spectra of astilbin with HSA. (∆λ = 15 nm); (B) Synchronous fluorescence spectra of astilbin with HSA. (∆λ = 60 nm).

## Sample Availability

Samples of the compounds are not available from the authors.
