# Supplementary material for: Molecular Insight into the Binding of Astilbin with Human Serum Albumin and Its Effect on Antioxidant Characteristics of Astilbin
Source: Molecules. 2022 Jul 13;27(14):4487. doi: 10.3390/molecules27144487 (PMC9321622; doi:10.3390/molecules27144487)
Supplement: Supplementary file 1 [file molecules-27-04487-s001.zip › molecules-1767936-supplementary.pdf]

Supplementary Materials

Molecular Insight into the Binding of Astilbin with Human Serum Albumin and Its Effect on Antioxidant Characteristics of Astilbin

Xiangyu Han [1,†], Jing Sun [1,2,†], Tianmei Niu [1,†], Beibei Mao [1], Shijie Gao [3], Pan Zhao [1,*] and Linlin Sun [1,3,*]

[1] College of Pharmacy, Shandong University of Traditional Chinese Medicine, Jinan 250355, China; hanxiangyujiayou@163.com (X.H.); 13065016960@163.com (J.S.); niutianmei123@163.com (T.N.); maobeibei89@163.com (B.M.)
[2] School of Chinese Materia Medica, Beijing University of Chinese Medicine, Beijing 100029, China
[3] Experimental Center, Shandong University of Traditional Chinese Medicine, Jinan 250355, China; gsj77@126.com
* Correspondence: 60030080@sdutcm.edu.cn (P.Z.); 60011923@sdutcm.edu.cn (L.S.)
† These authors contributed equally to this work.

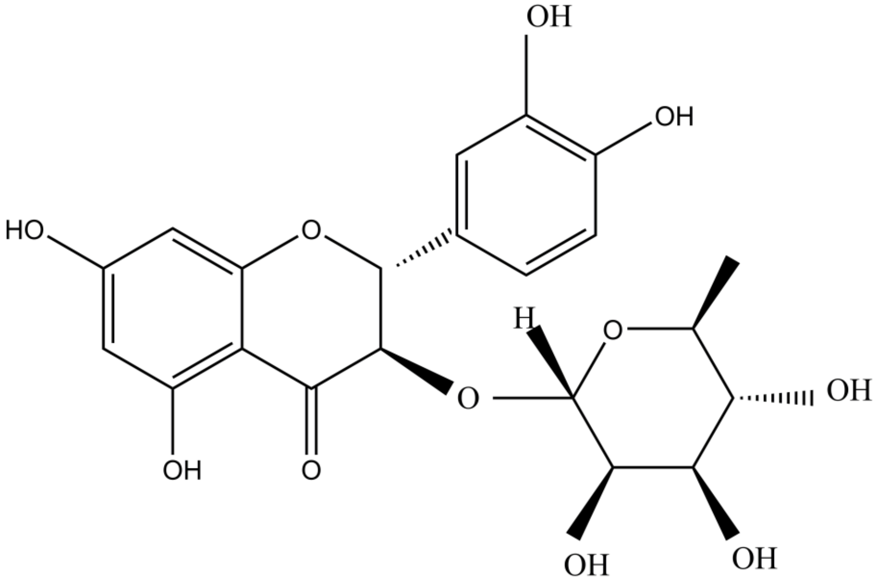

Figure S1. The Chemical structure of astilbin.

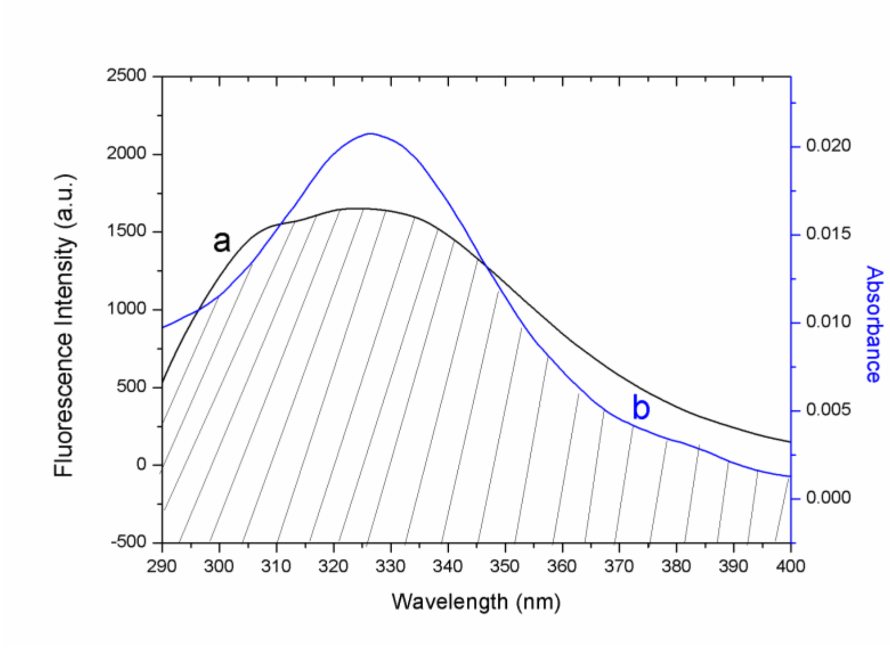

Figure S2: Overlap of the fluorescence emission spectrum of HSA with the UV-Vis absorption of astilbin. (a) is the fluorescence emission spectrum of HSA, C_{HSA}= 2.5 × 10^{-6} mol·L^{-1}; (b) is the UV-Vis absorption of astilbin, the C_{HSA}=2.5 × 10^{-6} mol·L^{-1}.

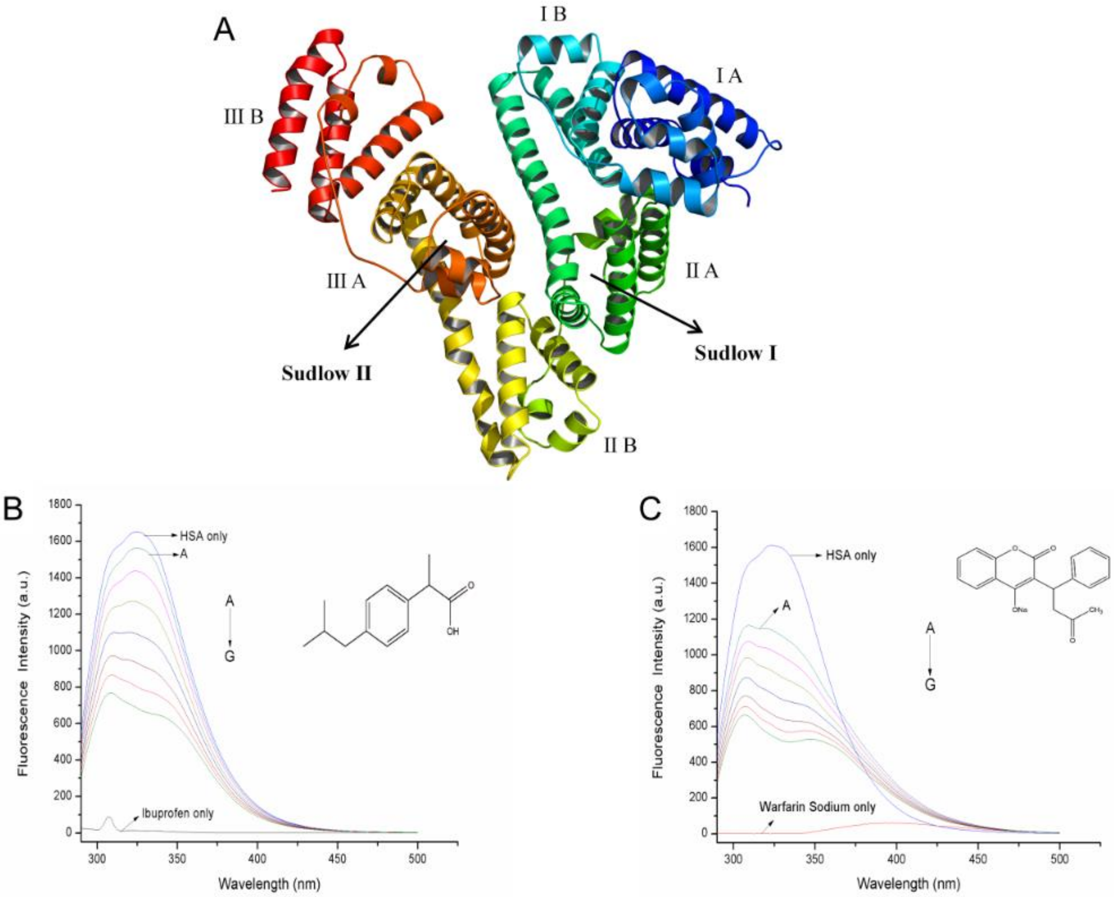

Figure S3: **A**: Structure of HSA showing the subdomains; **B–C**: Effects of different site competitors on HSA–astilbin complex; $C_{HSA} = C_{Warfarin\ sodium} = C_{Ibuprofen}$=2.5 × 10^{-6}mol·L^{-1}; $C_{astilbin}$(A→G):0, 1.25, 2.5, 5, 7.5, 10, 12.5 × 10^{-6}mol·L^{-1}. Temperature was 298 K. pH was 7.4 (Tris-HCl)

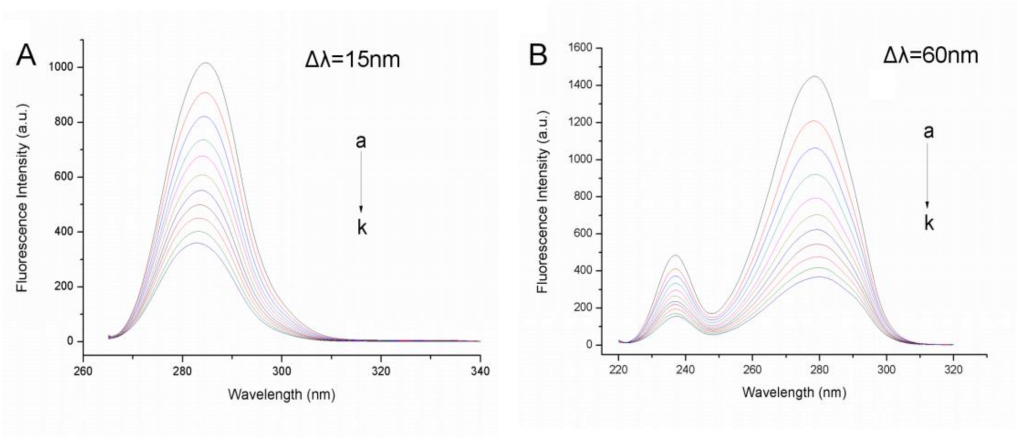

Figure S4: **A**: Synchronous fluorescence spectra of astilbin with HSA. (Δλ = 15 nm);

B: Synchronous fluorescence spectra of astilbin with HSA. (Δλ = 60 nm);
